# Supplementary material for: Microenvironmental arginine restriction sensitizes pancreatic cancers to polyunsaturated fatty acids by suppression of lipid synthesis
Source: eLife. 2026 Aug 5;14:RP106492. doi: 10.7554/eLife.106492 (PMC13441298; doi:10.7554/eLife.106492)
Supplement: Supplementary file 3 [file elife-106492-fig1-figsupp3-data1.zip › 1K_Supplement1_3A_Annotated.pdf]

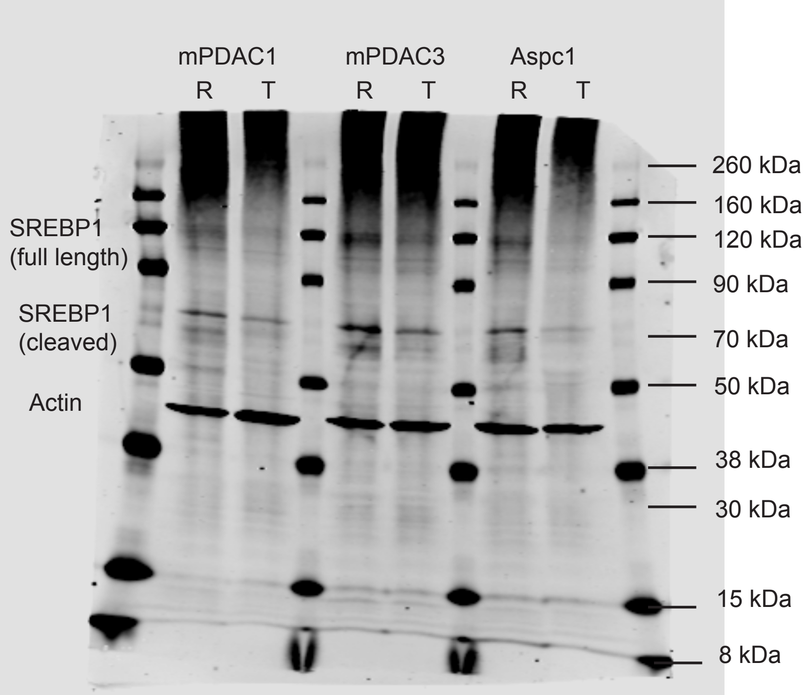
mPDAC1
mPDAC3
Aspc1
R
T
R
T
R
T
SREBP1
(full length)
SREBP1
(cleaved)
Actin
260 kDa
160 kDa
120 kDa
90 kDa
70 kDa
50 kDa
38 kDa
30 kDa
15 kDa
8 kDa

T: TIFM

R: RPMI
