# Supplementary material for: Microenvironmental arginine restriction sensitizes pancreatic cancers to polyunsaturated fatty acids by suppression of lipid synthesis
Source: eLife. 2026 Aug 5;14:RP106492. doi: 10.7554/eLife.106492 (PMC13441298; doi:10.7554/eLife.106492)
Supplement: Supplementary file 5 [file elife-106492-fig1-figsupp3-data3.zip › Figure 1 - Source data 4/1N_Annotated.pdf]

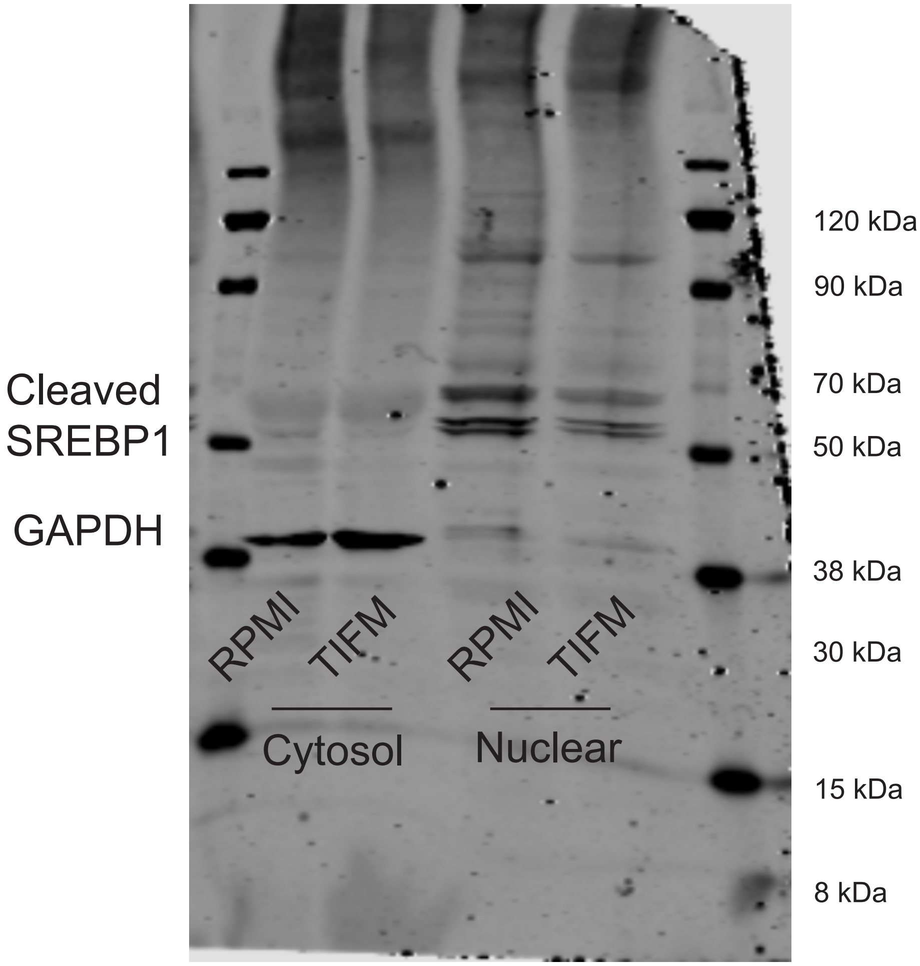
Cleaved
SREBP1
GAPDH
RPMI
TIFM
RPMI
TIFM
Cytosol
Nuclear
120 kDa
90 kDa
70 kDa
50 kDa
38 kDa
30 kDa
15 kDa
8 kDa

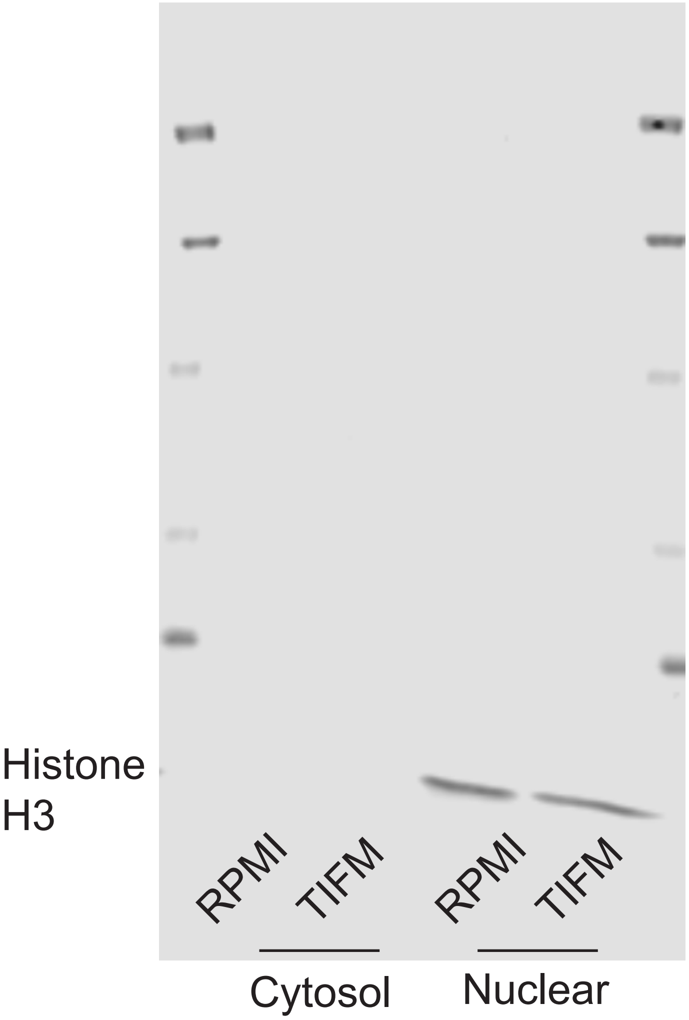
Histone
H3
RPMI
TIFM
RPMI
TIFM
Cytosol
Nuclear
