# Supplementary material for: Microenvironmental arginine restriction sensitizes pancreatic cancers to polyunsaturated fatty acids by suppression of lipid synthesis
Source: eLife. 2026 Aug 5;14:RP106492. doi: 10.7554/eLife.106492 (PMC13441298; doi:10.7554/eLife.106492)
Supplement: Supplementary file 8 [file elife-106492-fig2-figsupp2-data1.zip › Figure 2 - Source data 6/Fig2_Supplement2A_Annotated.pdf]

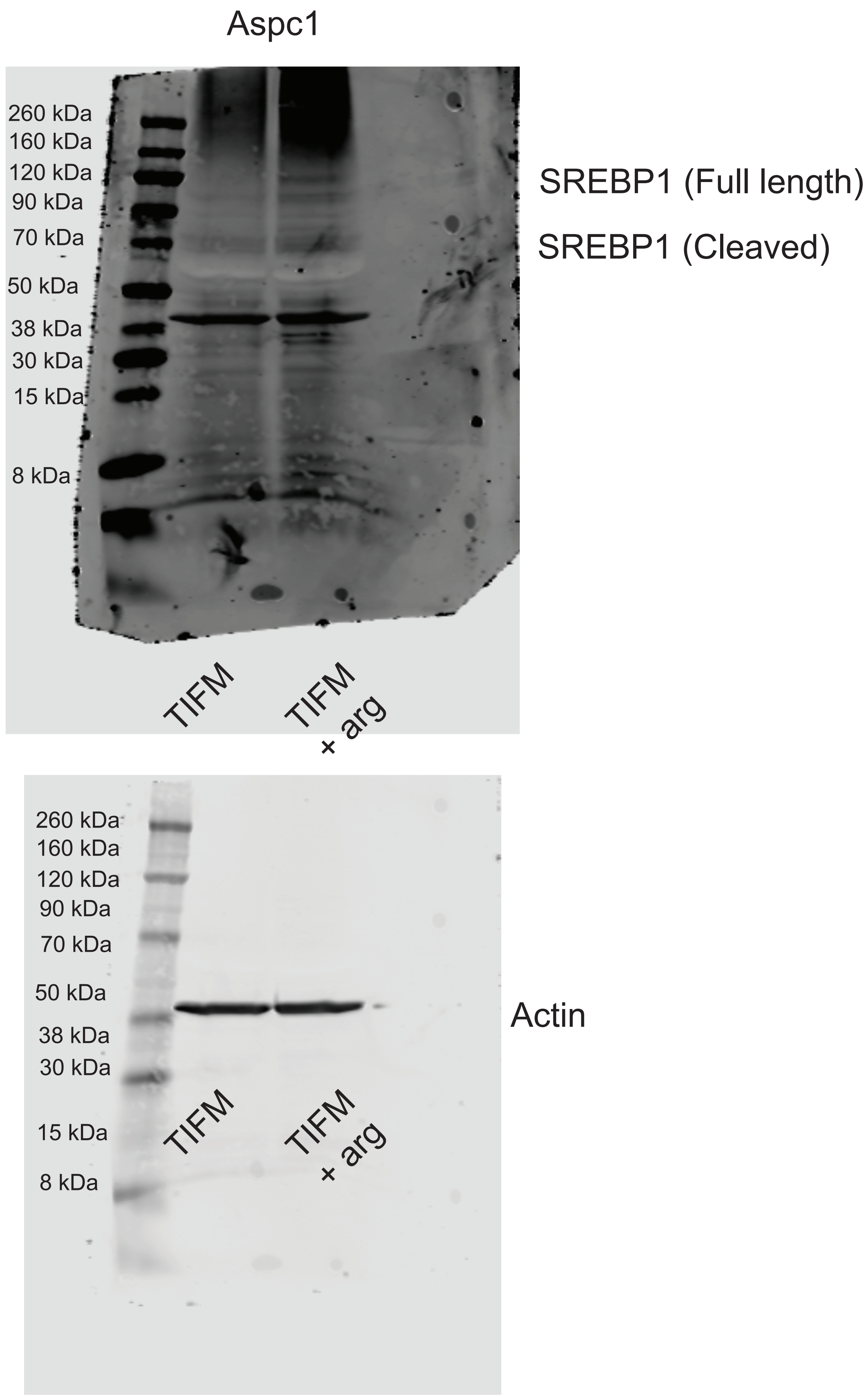

Aspc1
260 kDa
160 kDa
120 kDa
90 kDa
70 kDa
50 kDa
38 kDa
30 kDa
15 kDa
8 kDa
SREBP1 (Full length)
SREBP1 (Cleaved)
TIFM
TIFM + arg
260 kDa
160 kDa
120 kDa
90 kDa
70 kDa
50 kDa
38 kDa
30 kDa
15 kDa
8 kDa
Actin
TIFM
TIFM + arg
