# Supplementary material for: Microenvironmental arginine restriction sensitizes pancreatic cancers to polyunsaturated fatty acids by suppression of lipid synthesis
Source: eLife. 2026 Aug 5;14:RP106492. doi: 10.7554/eLife.106492 (PMC13441298; doi:10.7554/eLife.106492)
Supplement: Supplementary file 10 [file elife-106492-fig3-data2.zip › Figure 3 - Source data 3/3D_annotated.pdf]

mPDAC1

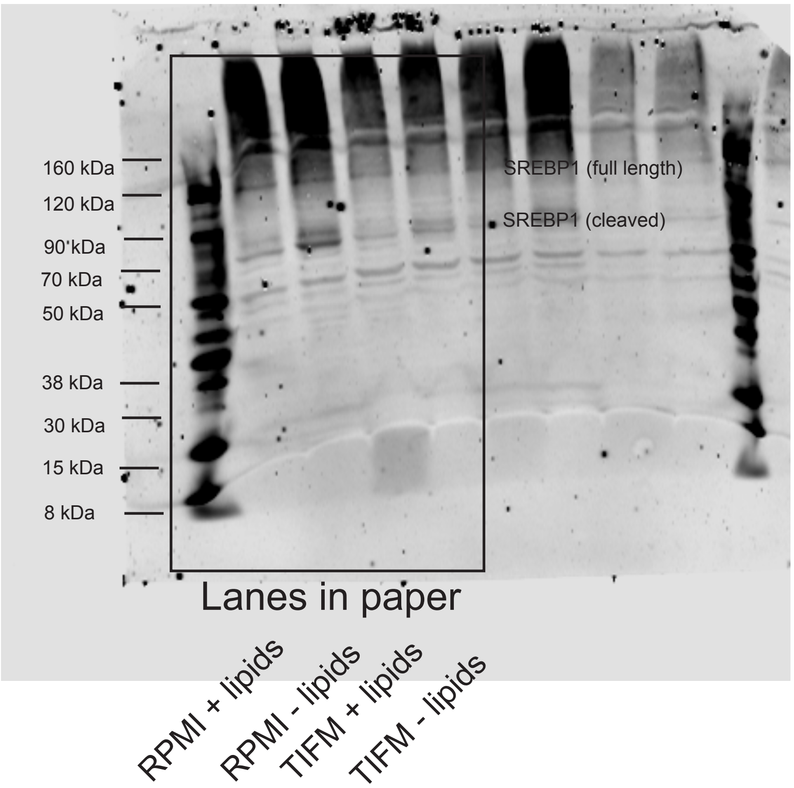
160 kDa
120 kDa
90 kDa
70 kDa
50 kDa
38 kDa
30 kDa
15 kDa
8 kDa
SREBP1 (full length)
SREBP1 (cleaved)
Lanes in paper
RPMI + lipids
RPMI - lipids
TIFM + lipids
TIFM - lipids

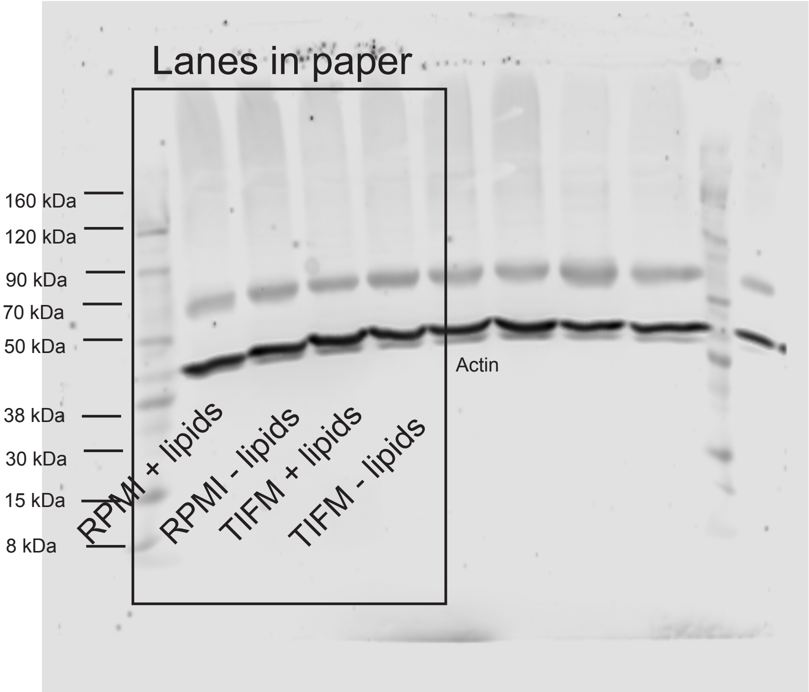
Lanes in paper
160 kDa
120 kDa
90 kDa
70 kDa
50 kDa
38 kDa
30 kDa
15 kDa
8 kDa
Actin
RPMI + lipids
RPMI - lipids
TIFM + lipids
TIFM - lipids
