# Supplementary material for: Microenvironmental arginine restriction sensitizes pancreatic cancers to polyunsaturated fatty acids by suppression of lipid synthesis
Source: eLife. 2026 Aug 5;14:RP106492. doi: 10.7554/eLife.106492 (PMC13441298; doi:10.7554/eLife.106492)
Supplement: Supplementary file 10 [file elife-106492-fig3-data2.zip › Figure 3 - Source data 3/3F_annotated.pdf]

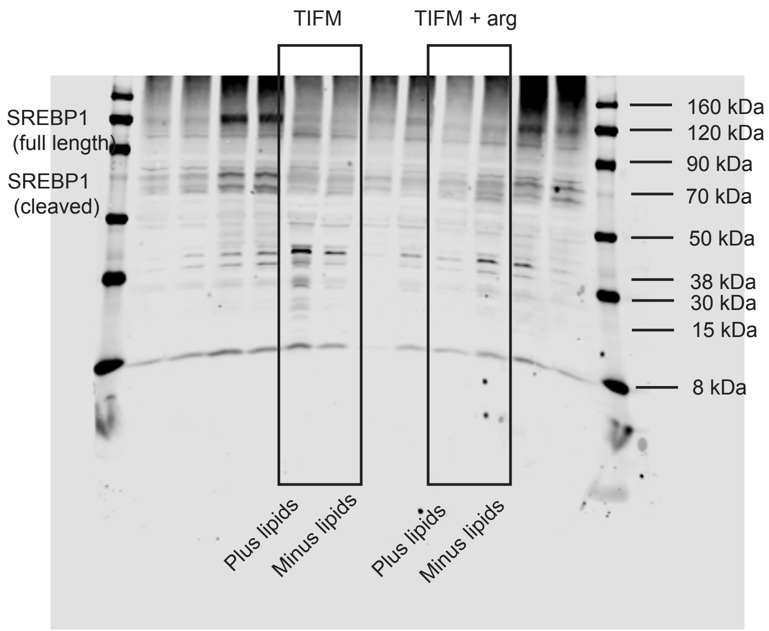

TIFM
TIFM + arg
SREBP1
(full length)
SREBP1
(cleaved)
160 kDa
120 kDa
90 kDa
70 kDa
50 kDa
38 kDa
30 kDa
15 kDa
8 kDa
Plus lipids
Minus lipids
Plus lipids
Minus lipids

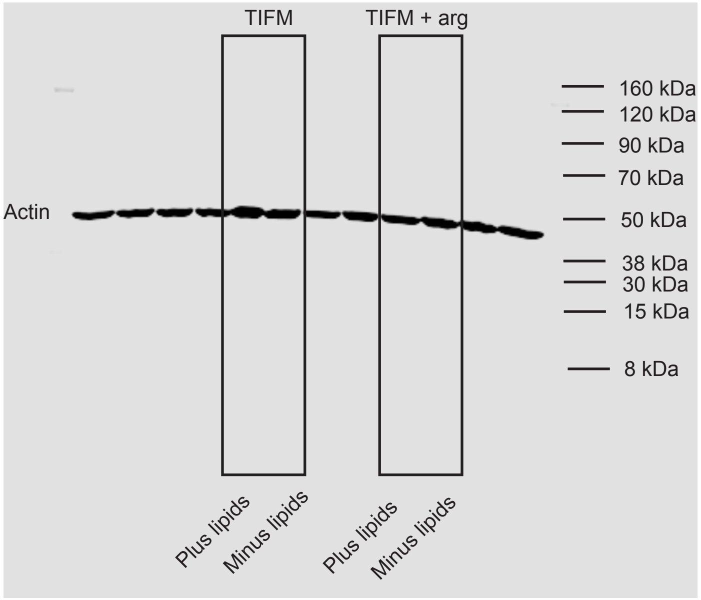

TIFM
TIFM + arg
Actin
160 kDa
120 kDa
90 kDa
70 kDa
50 kDa
38 kDa
30 kDa
15 kDa
8 kDa
Plus lipids
Minus lipids
Plus lipids
Minus lipids
