# Supplementary material for: Microenvironmental arginine restriction sensitizes pancreatic cancers to polyunsaturated fatty acids by suppression of lipid synthesis
Source: eLife. 2026 Aug 5;14:RP106492. doi: 10.7554/eLife.106492 (PMC13441298; doi:10.7554/eLife.106492)

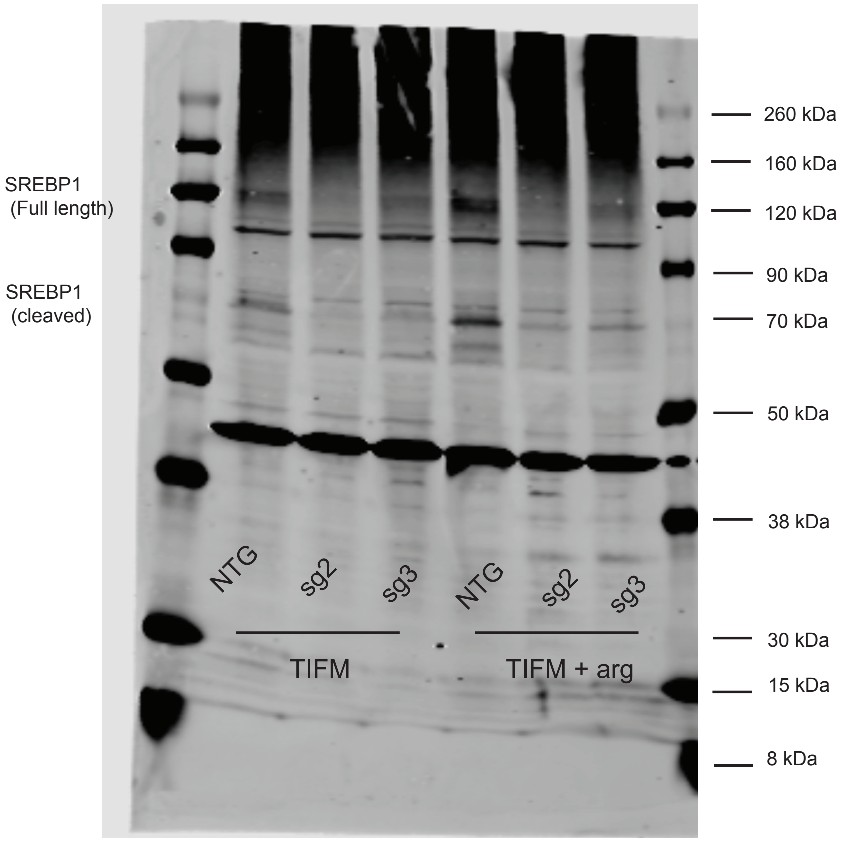

Sg2/3 targeting SREBP1

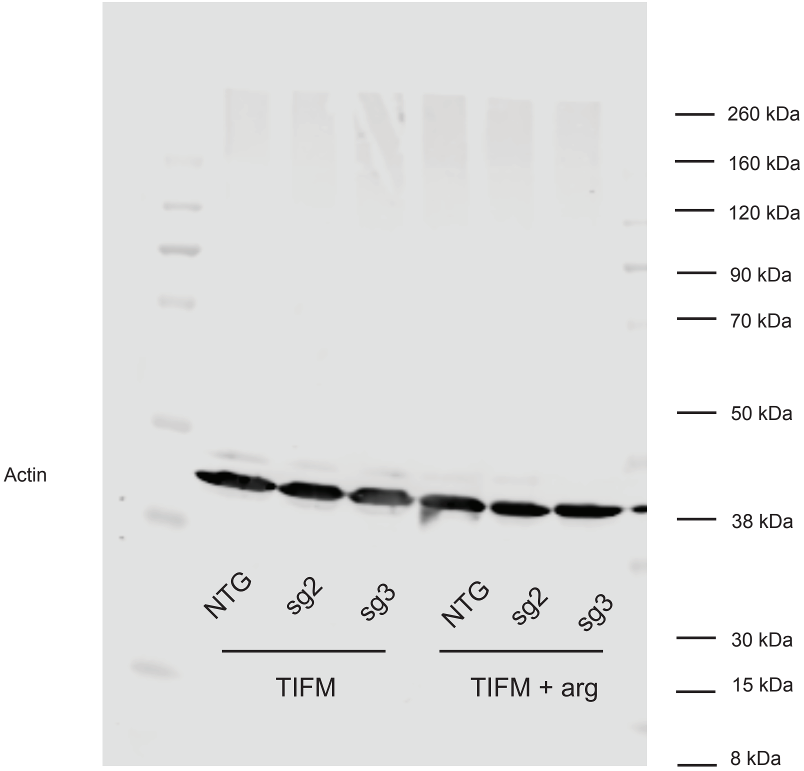

Supplement: Supplementary file 10 [file elife-106492-fig3-data2.zip › Figure 3 - Source data 3/3O_annotated.pdf]
