# Supplementary material for: Microenvironmental arginine restriction sensitizes pancreatic cancers to polyunsaturated fatty acids by suppression of lipid synthesis
Source: eLife. 2026 Aug 5;14:RP106492. doi: 10.7554/eLife.106492 (PMC13441298; doi:10.7554/eLife.106492)
Supplement: Supplementary file 12 [file elife-106492-fig3-figsupp2-data1.zip › Figure 3 - Source data 6/Fig3_Supplement2A_B_annotated.pdf]

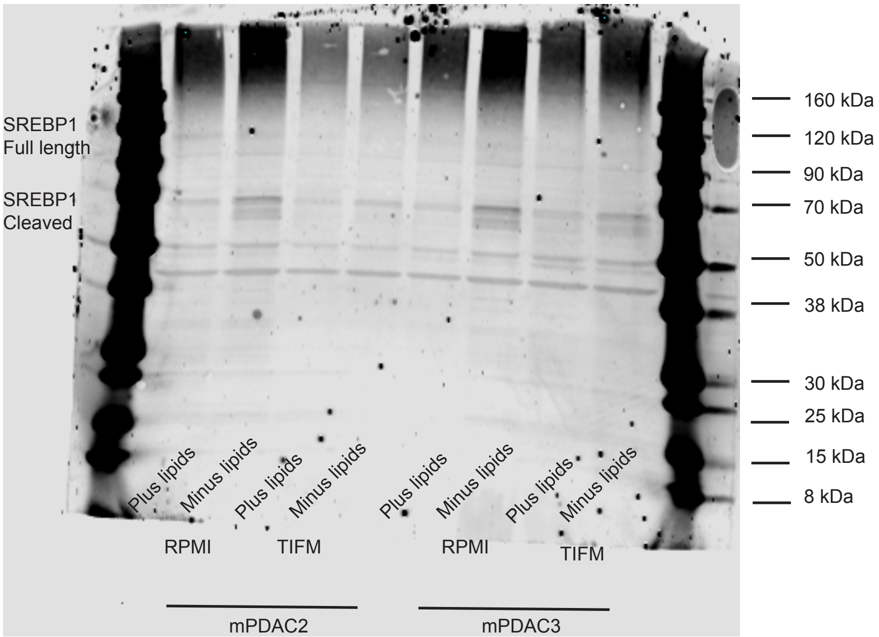
SREBP1
Full length
SREBP1
Cleaved
Plus lipids
Minus lipids
Plus lipids
Minus lipids
Plus lipids
Minus lipids
Plus lipids
Minus lipids
RPMI
TIFM
RPMI
TIFM
mPDAC2
mPDAC3
160 kDa
120 kDa
90 kDa
70 kDa
50 kDa
38 kDa
30 kDa
25 kDa
15 kDa
8 kDa

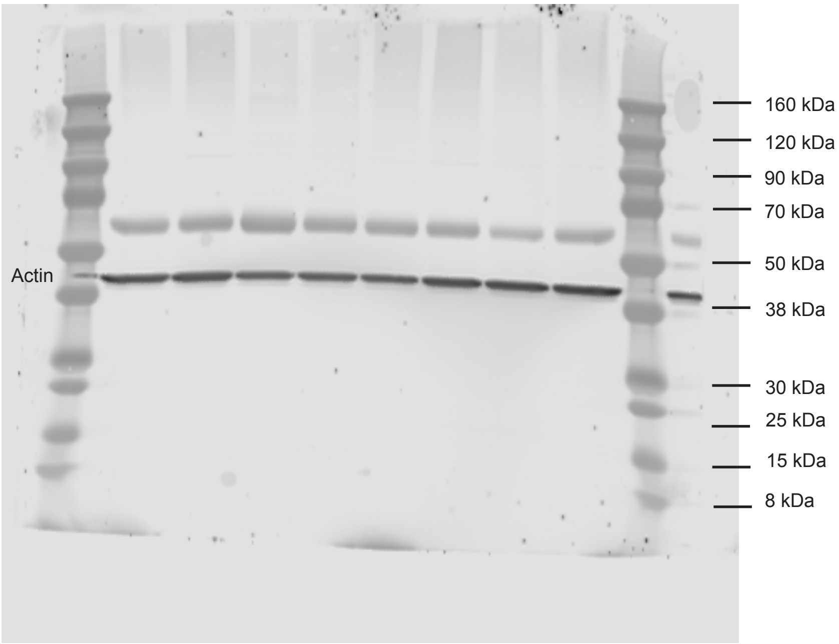
Actin
160 kDa
120 kDa
90 kDa
70 kDa
50 kDa
38 kDa
30 kDa
25 kDa
15 kDa
8 kDa
