# Supplementary material for: Microenvironmental arginine restriction sensitizes pancreatic cancers to polyunsaturated fatty acids by suppression of lipid synthesis
Source: eLife. 2026 Aug 5;14:RP106492. doi: 10.7554/eLife.106492 (PMC13441298; doi:10.7554/eLife.106492)
Supplement: Supplementary file 14 [file elife-106492-fig3-figsupp4-data1.zip › Figure 3 - Source data 8/Fig3_Supplement4A_RPMI_Annotated.pdf]

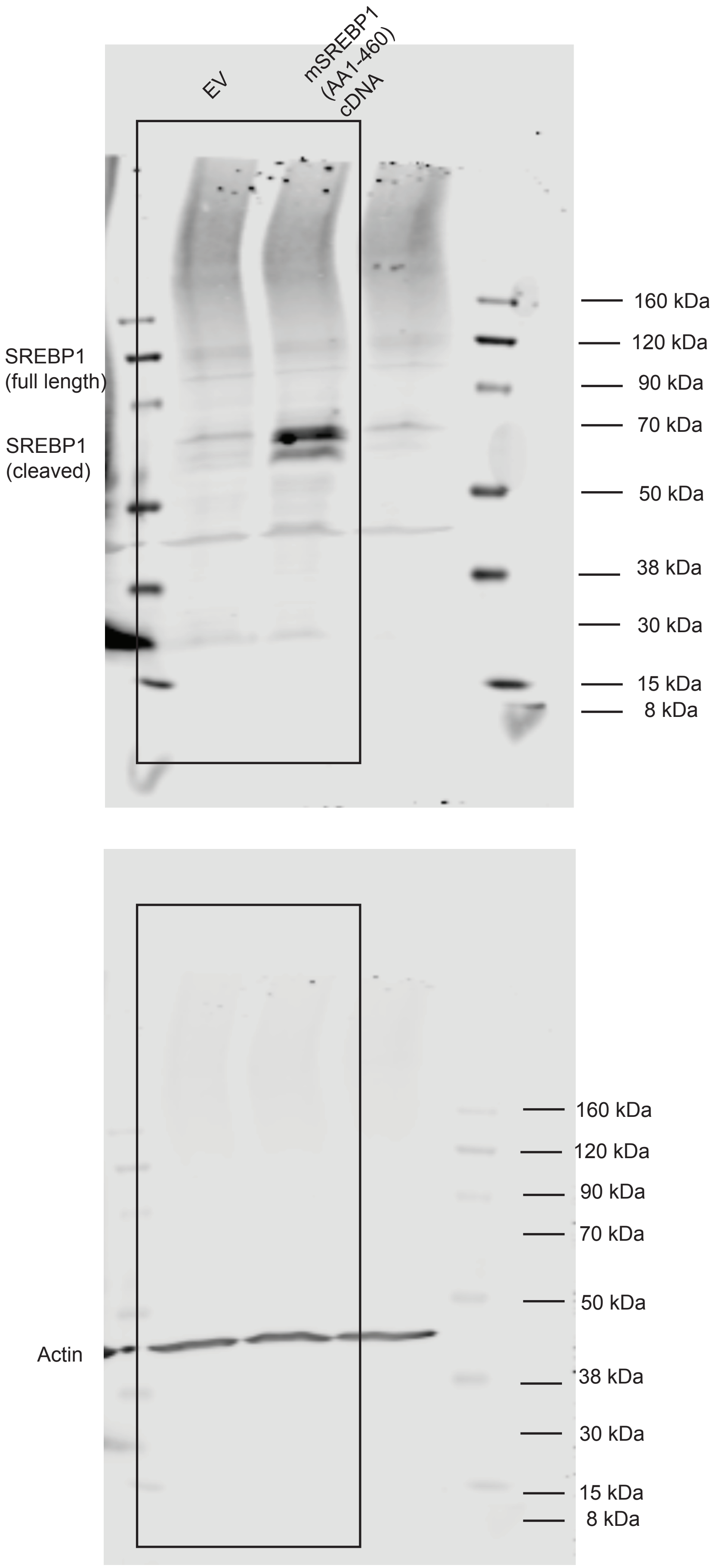
EV
mSREBP1 (AA1-460) cDNA
SREBP1 (full length)
SREBP1 (cleaved)
160 kDa
120 kDa
90 kDa
70 kDa
50 kDa
38 kDa
30 kDa
15 kDa
8 kDa
Actin
160 kDa
120 kDa
90 kDa
70 kDa
50 kDa
38 kDa
30 kDa
15 kDa
8 kDa
