# Supplementary material for: Microenvironmental arginine restriction sensitizes pancreatic cancers to polyunsaturated fatty acids by suppression of lipid synthesis
Source: eLife. 2026 Aug 5;14:RP106492. doi: 10.7554/eLife.106492 (PMC13441298; doi:10.7554/eLife.106492)
Supplement: Supplementary file 16 [file elife-106492-fig4-data1.zip › Figure 4 - Source data 1/4C_annotated.pdf]

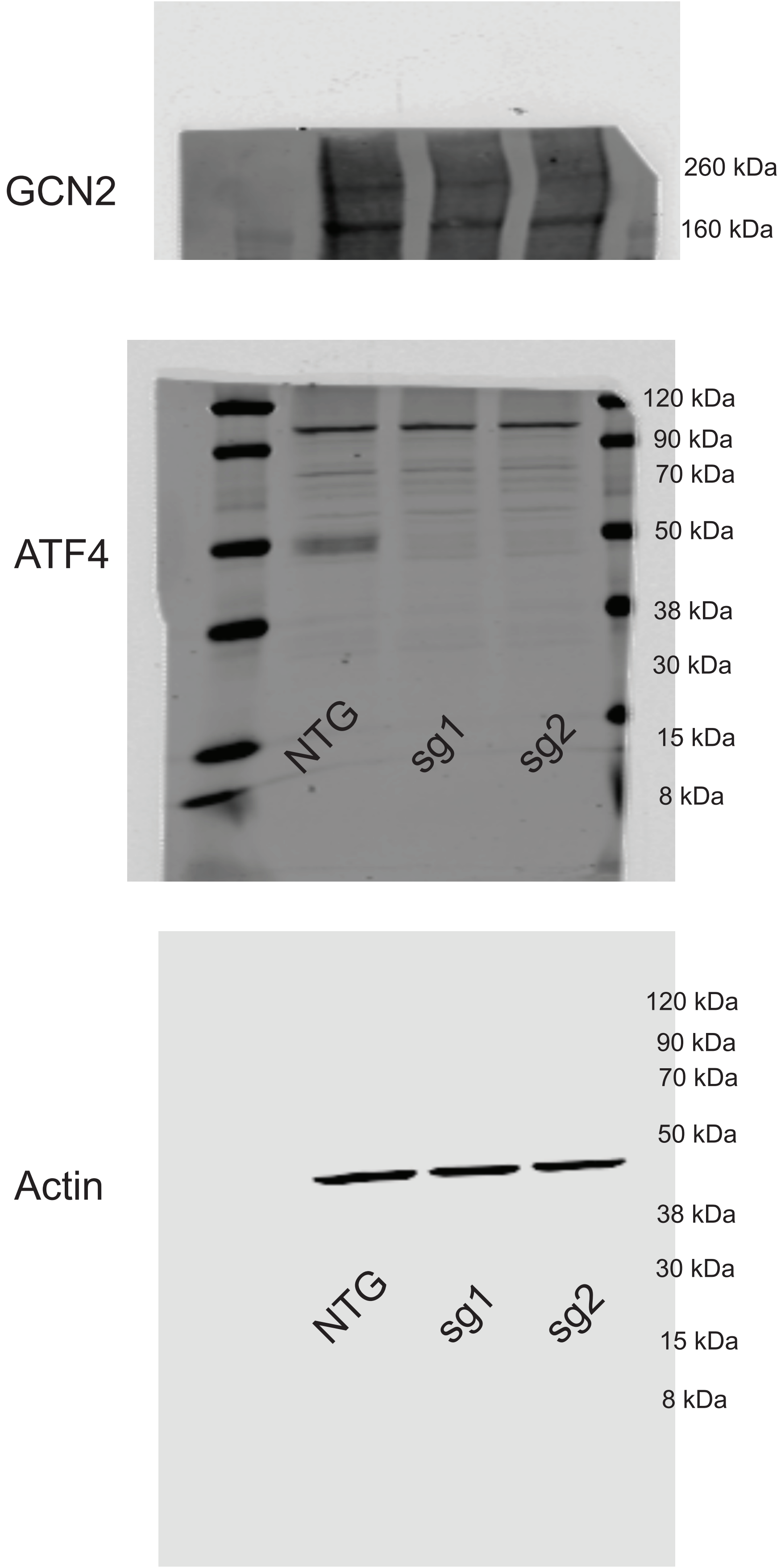
GCN2
260 kDa
160 kDa
ATF4
120 kDa
90 kDa
70 kDa
50 kDa
38 kDa
30 kDa
15 kDa
8 kDa
NTG
sg1
sg2
Actin
120 kDa
90 kDa
70 kDa
50 kDa
38 kDa
30 kDa
15 kDa
8 kDa
NTG
sg1
sg2
