# Supplementary material for: Microenvironmental arginine restriction sensitizes pancreatic cancers to polyunsaturated fatty acids by suppression of lipid synthesis
Source: eLife. 2026 Aug 5;14:RP106492. doi: 10.7554/eLife.106492 (PMC13441298; doi:10.7554/eLife.106492)
Supplement: Peer review history

# Editor report: eLife Assessment

**DOI:** 10.7554/eLife.106492.3.sa0

**Authors:** Ivan Topisirovic

**Affiliations:** McGill University Canada

**Keywords:** Compelling, Important

This **important** study demonstrates that nutrient stress engenders metabolic vulnerabilities in pancreatic ductal adenocarcinoma (PDAC). By combining cell line and mouse models, the authors provide **compelling** evidence showing that arginine depletion from the microenvironment disrupts lipid homeostasis in PDAC resulting in ferroptosis upon exposure of tumors to polyunsaturated fatty acids. This report is likely to be of broad interest to researchers interested in studying cancer biology, metabolic adaptations and stress responses.

# Referee report: Reviewer #1 (Public review)

**DOI:** 10.7554/eLife.106492.3.sa1

Summary:

In this study, the authors set out to define how arginine availability regulates lipid metabolism and to explore the implications of this relationship in pancreatic ductal adenocarcinoma (PDAC), a tumor type known to exist in an arginine-poor microenvironment. Using a combination of rigorous genetic and metabolomic approaches, they uncover a previously underappreciated role for arginine in maintaining lipid homeostasis. Importantly, they demonstrate that arginine deprivation sensitizes PDAC cells to ferroptosis through lipidome perturbations, which can be exploited therapeutically via co-treatment with aESA and ferroptosis inducers (FINs). These findings have meaningful implications for the field. They not only shed light on the metabolic vulnerabilities created by nutrient restriction in PDAC, but also suggest a practical avenue for combination therapies that exploit ferroptosis sensitivity. This is particularly relevant in the context of pancreatic cancer, which is notoriously resistant to conventional treatments. The methods employed are broadly applicable to other nutrient-stress contexts and may inspire similar investigations in other solid tumor types.

Strengths:

One of the major strengths of the study is the use of complementary and well-controlled approaches-including metabolomic profiling, genetic perturbations, and in vivo models-to support the central hypothesis. The experiments are thoughtfully designed and clearly presented, and the conclusions are, for the most part, well supported by the data. The findings provide mechanistic insight into nutrient-lipid crosstalk and identify a potential therapeutic strategy for targeting arginine-deprived tumors.

Comments on revised version:

The authors have substantially strengthened the revised manuscript and have addressed my prior concerns, and the evidence supports the central conclusions. This work provides meaningful insight into how nutrient limitation in the tumor microenvironment creates metabolic liabilities that may be therapeutically exploited, and it should be of interest to investigators studying cancer metabolism, pancreatic cancer, lipid biology, and ferroptosis.

# Referee report: Reviewer #2 (Public review)

**DOI:** 10.7554/eLife.106492.3.sa2

This study by Jonker et al., examines how the metabolic adaptations to the microenvironment by pancreatic ductal adenocarcinomas (PDAC) present vulnerabilities that could be used for therapeutic purposes. The evidence supporting the claims of the authors is mostly solid, and the multiplicity of models used, as well as the combination of in vitro and in vivo work are appreciated, but some conclusions would benefit from additional substantiation. This work would be of interest to biologists working on the impact of microenvironment and metabolism in cancer, and especially those investigating pancreatic cancer.

In this study, the authors use mostly "doublings per day" as an indicator of cell death, notably for figures 4 to 6. However, proliferative arrest (or a decrease in the proliferative rate) is not necessarily synonymous with cell death. It might be nice to complement these experiments with a true measure of cell death (e.g. PI uptake).

# Referee report: Reviewer #3 (Public review)

**DOI:** 10.7554/eLife.106492.3.sa3

This important study investigates the impact of nutrient stress in the tumor microenvironment (TME), focusing on lipid metabolism in pancreatic ductal adenocarcinoma (PDAC). Understanding TME composition is crucial, as it highlights cancer vulnerabilities independent of intracellular mutations, particularly because PDAC tumors are often exposed to limited nutrient availability due to reduced perfusion.

By utilizing a medium that mimics the nutrient conditions of PDAC tumors, the authors convincingly show that TME nutrient stress suppresses SREBP1, leading to reduced lipid synthesis, with low arginine levels identified as a key driver of this suppression. Importantly, mice with arginine-starved pancreatic tumors respond to polyunsaturated fatty acid-rich diet. This discovery uncovers a synthetic lethal interaction in the tumor microenvironment that could be leveraged through dietary interventions.

Comments on revised version:

The authors have satisfactorily resolved all previously raised concerns through the inclusion of additional data and clarifications in the discussion.

# Author comment: Author response

**DOI:** 10.7554/eLife.106492.3.sa4

**Authors:** Patrick B Jonker, Mumina Sadullozoda, Guillaume Cognet, Juan J Apiz Saab, Kelly H Sokol, Violet X Wu, Deepa Kumari, Colin Sheehan, Mete E Ozgurses, Darby Agovino, Grace Croley, Lindsey N Dzierozynski, Chufan Cai, Leah M Ziolkowski, Smit A Patel, Althea Bock-Hughes, Kay F Macleod, Hardik Shah, Jonathan L Coloff, Evan C Lien, Alexander Muir

**Affiliations:** University of Chicago Chicago United States; University of Chicago Chicago United States; University of Chicago Chicago United States; University of Chicago Chicago United States; Van Andel Institute Grand Rapids United States; University of Chicago Chicago United States; University of Chicago Chicago United States; University of Chicago Chicago United States; University of Illinois Chicago Chicago United States; University of Chicago Chicago United States; University of Chicago Chicago United States; University of Chicago Chicago United States; Dana-Farber Cancer Institute Boston United States; University of Chicago Chicagi United States; University of Chicago Chicago United States; University of Chicago Chicago United States; University of Chicago Chicago United States; University of Chicago Chicago United States; University of Illinois Chicago Chicago United States; Van Andel Institute Grand Rapids United States; University of Chicago Chicago United States

The following is the authors’ response to the original reviews.

**Public Reviews:**

**Reviewer #1 (Public review):**

Summary:

In this study, the authors set out to define how arginine availability regulates lipid metabolism and to explore the implications of this relationship in pancreatic ductal adenocarcinoma (PDAC), a tumor type known to exist in an arginine-poor microenvironment. Using a combination of rigorous genetic and metabolomic approaches, they uncover a previously underappreciated role for arginine in maintaining lipid homeostasis. Importantly, they demonstrate that arginine deprivation sensitizes PDAC cells to ferroptosis through lipidome perturbations, which can be exploited therapeutically via co-treatment with aESA and ferroptosis inducers (FINs). These findings have meaningful implications for the field. They not only shed light on the metabolic vulnerabilities created by nutrient restriction in PDAC, but also suggest a practical avenue for combination therapies that exploit ferroptosis sensitivity. This is particularly relevant in the context of pancreatic cancer, which is notoriously resistant to conventional treatments. The methods employed are broadly applicable to other nutrient-stress contexts and may inspire similar investigations in other solid tumor types.

Strengths:

One of the major strengths of the study is the use of complementary and well-controlled approaches-including metabolomic profiling, genetic perturbations, and in vivo models-to support the central hypothesis. The experiments are thoughtfully designed and clearly presented, and the conclusions are, for the most part, well supported by the data. The findings provide mechanistic insight into nutrient-lipid crosstalk and identify a potential therapeutic strategy for targeting arginine-deprived tumors.

We thank the reviewer for their positive assessment of our manuscript.

Weaknesses:

A key weakness of the study lies in the mechanistic connection between arginine levels and SREBP1 activation. While the authors show that arginine restriction leads to reduced SREBP1 expression, the magnitude of this effect appears modest relative to the substantial changes observed in the lipidome. The study would benefit from a deeper analysis of SREBP1 regulation-particularly whether nuclear translocation or activation is affected. This could be addressed by examining the nuclear pool of SREBP1, using either subcellular fractionation or improved immunofluorescence imaging in both cell lines and tissue samples.

We thank the reviewer for this comment and in our revised manuscript have undertaken several new studies to assess how the nuclear pool of SREBP1 is regulated by arginine starvation. We further identified one mechanism by which arginine starvation suppresses SREBP1 protein levels, namely GCN activation. We believe these additional studies strengthen the manuscript and appreciate the reviewer suggesting these studies.

Another area where additional context would strengthen the manuscript is in the transcriptomic profiling of PDAC cells cultured in a tumor interstitial fluid mimic (TIFM). While the study emphasizes lipid-related pathways, highlighting the most significantly upregulated and downregulated pathways in Figure 1B would give readers a broader perspective on how arginine restriction reprograms the PDAC transcriptome. For instance, because polyamines are downstream of arginine and are known to influence lipid metabolism, it would be worth discussing whether these metabolites contribute to the phenotypes observed. Similarly, an evaluation of whether Dgat1/2 expression is altered could help delineate the full scope of lipid metabolic rewiring.

We thank the reviewer for suggesting this change to our manuscript and we now provide much more extensive analysis of our transcriptomic analyses in Figure 1 – Figure supplement 1, which we think will make our manuscript more useful to readers.

Finally, it is worth noting that the KPC mouse model used in this study is based on conditional deletion of p53, which leads to faster-growing tumors and a distinct tumor microenvironment compared to models harboring the p53^R172H point mutation. Including a brief discussion of this distinction would help readers contextualize the translational relevance of the findings.

We have revised the manuscript to include a discussion of this point.

**Reviewer #2 (Public review):**

This study by Jonker et al. examines how the metabolic adaptations to the microenvironment by pancreatic ductal adenocarcinomas (PDAC) present vulnerabilities that could be used for therapeutic purposes. The evidence supporting the claims of the authors is mostly solid, and the multiplicity of models used, as well as the combination of in vitro and in vivo work, are appreciated, but some conclusions would benefit from additional substantiation. This work would be of interest to biologists working on the impact of microenvironment and metabolism in cancer, and especially those investigating pancreatic cancer.

We thank the reviewer for their positive assessment of our manuscript.

In this study, the authors use mostly "doublings per day" as an indicator of cell death, notably for Figures 4 to 6. However, proliferative arrest (or a decrease in the proliferative rate) is not necessarily synonymous with cell death. It might be nice to complement these experiments with a true measure of cell death (e.g., PI uptake).

We thank the reviewer for this important comment and have performed extensive additional experiments to measure cell death directly via viability markers in addition to our indirect measurements of cell number at the start and end of experiments. We believe these additions strengthen our claims that PUFAs cause arginine starved PDAC cells to undergo ferroptotic cell death.

The composition of Tumor Interstitial Fluid Medium (TIFM) was published previously, but nonetheless a reminder of the composition of this medium in a Supplemental file of this study might be helpful. In particular, at the start of the Results section, the nature of serum/lipids in the different media should be specifically noted, especially given that the subsequent focus of the work is on lipids/SREBP. It is known that differences in the extracellular availability of lipids can profoundly alter de novo lipid biosynthesis pathways.

We thank the reviewer for this comment. We have edited the text to provide additional context on the composition of TIFM, especially lipid availability. We further have provided a supplemental file with the composition of TIFM. We hope this will make the manuscript more useful and readily interpretable for readers.

**Reviewer #3 (Public review):**

This important study investigates the impact of nutrient stress in the tumor microenvironment (TME), focusing on lipid metabolism in pancreatic ductal adenocarcinoma (PDAC).

Understanding TME composition is crucial, as it highlights cancer vulnerabilities independent of intracellular mutations, particularly because PDAC tumors are often exposed to limited nutrient availability due to reduced perfusion.

By utilizing a medium that mimics the nutrient conditions of PDAC tumors, the authors convincingly show that TME nutrient stress suppresses SREBP1, leading to reduced lipid synthesis, with low arginine levels identified as a key driver of this suppression. Importantly, mice with arginine-starved pancreatic tumors respond to a polyunsaturated fatty acid-rich diet. This discovery uncovers a synthetic lethal interaction in the tumor microenvironment that could be leveraged through dietary interventions.

The conclusions of this paper are mostly well supported by data; however, below are some aspects that could be further clarified.

We thank the reviewer for their positive assessment of our manuscript.

This study uses PDAC cells from the LSL-Kras G12D/+ ; Trp53 ; Pdx-1-Cre PDAC model. The authors convincingly demonstrate that the cell-extrinsic stimuli of low arginine availability suppress lipid synthesis and thus exert a dominant effect over the cell-intrinsic oncogenic Ras mutation, which is known to enhance fatty acid synthesis. Could the effect of low arginine on lipid synthesis be specific for certain mutations in PDAC? It would be interesting to investigate or discuss whether different mutations show the same SREBP1 reduction caused by low arginine levels, and whether these low SREBP1 levels can be ameliorated by arginine re-supplementation. Here, Jonker et al. show that human PDAC cells cultured in TIFM have reduced SREBP1 levels (Figure 1 - Figure supplement 1C). It would be further supportive of their conclusions if the authors could show that arginine re-supplementation is sufficient to restore SREBP1 levels in human PDAC cells.

We thank the reviewer for this comment. In response, we have now shown that arginine supplementation increases SREBP1 levels and fatty acid synthesis in human PDAC cells (Figure 2 – Figure supplement 2). Further, we have also updated the manuscript to discuss that using the LSL-Kras G12D/+; Trp53; Pdx-1-Cre PDAC model limits our ability to assess how genetic differences influence the response to arginine starvation. We additionally discuss the genetic diversity of the human PDAC cell lines used in these studies, which do include different oncogenic mutations. We believe that these results provide some data that the findings we have made regarding arginine deprivation and SREBP in our genetically defined murine PDAC cell line are applicable to human PDAC cells with more diverse oncogenic lesions.

The authors demonstrate that mPDAC cells cultured in RPMI and subsequently implanted into an orthotopic mouse model exhibit reduced expression of SREBP target genes when compared to in vitro cultured mPDAC-RPMI cells. This finding is in line with the observation that culturing PDAC cells in TIFM downregulates SREBP target genes compared to PDAC cells cultured in RPMI. However, caution is needed when directly comparing mPDAC-RPMI cultured cells to those in the orthotopic model, as the latter may include non-tumor cells and additional factors that could confound the results. The authors should explicitly acknowledge this limitation in their study.

We thank the reviewer for this important caveat and we have revised to text to address this point. Importantly, we note that for all comparisons between in vitro and in vivo cultures, we carefully sort malignant cancer cells from orthotopic tumors prior to analysis. We believe this approach mitigates the impact of stromal contamination on our analyses.

The in vivo evidence demonstrating that PUFA-rich tung oil reduces tumor size is compelling. However, the specific in vitro findings regarding its impact on doubling rates per day, particularly in the context of arginine-dependent PUFA supplementation, require further explanation. To enhance the robustness of their data and conclusions, the authors could consider conducting additional cell viability and proliferation assays. Moreover, it would be valuable to assess whether the observed effects on doubling rates per day remain significant after normalizing the data to the initial doubling time prior to PUFA supplementation. This is in particular important regarding the statement that "Addition of arginine significantly decreases sensitivity to a-ESA" as these cells already start with a higher doubling rate prior to a-ESA treatment.

We thank the reviewer for this important comment and have performed additional experiments to measure cell death directly via viability markers in addition to our indirect measurements of cell number at the start and end of experiments. Furthermore, to address the issue of different rates of cell growth in cultures affecting the response to perturbations, we also used growth rate corrected metrics (PMID: 27135972) to ensure that affects of perturbations on cell growth and viability are not confounded by the baseline proliferative kinetics of the cells under various media conditions. We believe these additions strengthen our claims that arginine starvation sensitizes PDAC cells to PUFAs.

Overall, this paper presents a compelling study that significantly enhances our understanding of the PDAC tumor microenvironment and its complex interactions with the tumor lipid metabolism.

We again thank the reviewer for their positive assessment of our manuscript.

**Recommendations for the authors:**

**Reviewer #1 (Recommendations for the authors):**

In this study, the authors employ rigorous genetic and biochemical (metabolomic) approaches to uncover a previously unappreciated role for arginine in regulating lipid homeostasis. They further demonstrate the relevance of this pathway in pancreatic tumors, a solid tumor type often characterized by limited access to extracellular arginine. The authors present compelling evidence that arginine deprivation creates a metabolic liability, rendering tumors more susceptible to lipidome perturbations. This vulnerability can be therapeutically exploited through co-treatment with aESA and FIN to induce ferroptosis. Overall, the conclusions are convincing, the manuscript is well-written, and the figures are clearly presented.

We again thank the reviewer for their positive assessment of our manuscript.

The key weakness of the study lies in the mechanistic link between arginine levels and SREBP1 expression. While the data support the authors' argument, the observed changes in SREBP1 expression following arginine restriction appear modest relative to the more pronounced changes in the lipidome. To strengthen this connection, the authors may consider performing cellular fractionation to focus their analysis on the nuclear (active) pool of SREBP1. Improved immunofluorescence imaging and quantification of nuclear SREBP1 levels in tissues would also provide additional support for their model.

We thank the reviewers for this helpful comment. To strengthen this study, we both examined the nuclear levels of SREBP1 in TIFM cultured cells and worked to identify the mechanistic link connecting arginine levels of SREBP1 expression.

First, we found that arginine starvation does not lead to nuclear exclusion of SREBP1. We believe this finding strengthens our conclusion that arginine starvation regulates SREBP1 at the level of protein expression. We do agree with the reviewer that the change in SREBP1 protein level is modest, but we do show the effects of arginine on PDAC cell lipid metabolism are SREBP1 dependent (Figure 3O-P, Figure 5F, Figure 5 – Figure supplement 2D). Thus, we interpret these data that even the relatively modest change in SREBP1 protein levels are sufficient to cause large changes in the output of this transcription factor and the cellular lipidome.

Second, we determined if the arginine-responsive GCN2 signaling pathway, which is known to regulate SREBP1, could contribute to the suppression of SREBP1 observed in PDAC cells. We found that GCN2 signaling is activated in PDAC cells in TIFM culture by arginine starvation and is active in animal tumors. We further found that activation of GCN2 is in part responsible for suppression of SREBP1, which is consistent with prior literature describing a role for GCN2 activation in suppressing SREBP1 translation (PMID: 17276353). Thus, while other mechanisms are at play in transducing arginine starvation to reduced SREBP1 protein levels, we have identified one mechanism (activation of GCN2) by which arginine starvation suppresses SREBP1, leading to the lipidomic changes we observed upon starvation of this amino acid.

In addition, it would be helpful for the authors to highlight the most significantly upregulated and downregulated pathways in Figure 1B to give a more comprehensive view of transcriptomic changes in PDAC cells cultured under TIFM conditions. For example, since polyamines are downstream of arginine and known to regulate lipid metabolism, could some of the observed effects be attributed to changes in polyamine levels? Similarly, do arginine levels affect the expression of Dgat1 or Dgat2?

We have added an additional Figure supplement to Figure 1 that include a comprehensive list of up- and downregulated gene sets in PDAC cells cultured in TIFM via GSEA analysis. We also added additional KEGG metabolic pathway analysis via GATOM (PMID: 35639928). We hope these additions will be useful for readers and point their attention to other metabolic pathways that are significantly altered by nutrient stress, such as the TCA cycle and oxidative phosphorylation, beyond those related to lipid metabolism that we investigated here.

From this analysis, we did not specifically note strong changes in the expression of polyamine metabolic enzymes or DGATs.

Finally, the KPC model used in this study involves conditional deletion of p53, which is known to produce tumors with a faster progression and a distinct tumor microenvironment compared to the more commonly used p53^R172H knock-in model. Including this point in the discussion would help contextualize the findings.

We thank the reviewers for mentioning this limitation of our study. In the results section of the test, we now included a discussion of the limitations of the mouse model used in the discussion of the work. We also highlight in the text now that in addition to our studies using the murine p53 deletion model that our studies make use of human PDAC lines that contain p53 mutations. We believe that these results provide some data that the findings we have made regarding arginine deprivation and SREBP in our genetically defined murine PDAC cell line are applicable to human PDAC cells with more diverse oncogenic lesions.

Minor comments to improve clarity:

(1) In Figure 3C, it would be helpful to annotate the PE-linked TG for clarity.

We do not understand exactly what PE-linked TGs refers to. We note in Fig. 3C that ether-linked triglycerides are labeled in orange and annotated as O-TG and vinyl ether-linked triglycerides are labeled in grey and annotated as P-TG.

(2) Is Figure 3P mislabeled? Both conditions are labeled as +Arg / -lipid.

We thank the reviewers for pointing out this mistake in the figure and have updated it to correctly label these samples as sgSREBP1 and sgNTG transduced PDAC cell lines.

**Reviewer #2 (Recommendations for the authors):**

(1) Figure 1B: Misspelling in Y axis "Normalized enrichment score".

We thank the authors for catching this mistake and have corrected this error.

(2) Figure 1B: Could the authors elaborate on why they decided to focus specifically on these three hits, which are not the most downregulated genes (the "top hits") appearing in the GSEA?

We chose to focus on lipid metabolism as multiple transcriptomic analysis tools, namely GSEA and GATOM, which specifically focuses on enrichment in KEGG annotated metabolic pathways, highlighted lipid synthesis as being the most transcriptionally regulated metabolic pathway in TIFM. To make this apparent to readers, we added an additional Figure supplement to Figure 1 that includes a comprehensive list of up- and downregulated gene sets in PDAC cells cultured in TIFM from GSEA and GATOM analysis. We hope these additions will make the logic for our focus on lipid synthesis clear and will be useful for readers in highlighting other metabolic pathways that are significantly altered by nutrient stress, such as the TCA cycle and oxidative phosphorylation.

(3) Figure 1: It might improve the clarity of the text if the three pairs of murine cell lines (mPDAC1, mPDAC2, mPDAC3) were introduced in a bit more detail in the main text and not just in the figure legend.

We have added more detail describing the three mouse cell lines used in the main text.

(4) Figure 1E: The authors may wish to comment on why they chose to perform transcriptomic analyses with the mPDAC3 derived models, and not mPDAC1 or mPDAC2, given that mPDAC3 appears to exhibit the most distinct phenotype of the three, according to the results presented in Figure 1 J-L.

The transcriptional analysis described in Fig. 1E was performed on a previously acquired dataset using mPDAC3 cell lines (PMID: 37254839), which is why this line was used. We have revised the text to make it clear that this transcriptional analysis uses pre-existing data from a previous publication.

(5) Figure 1L: The authors may wish to clarify why they only show relative palmitate to assess global fatty acid biosynthesis in these cell lines. There is a decrease in labeled palmitate of mPDAC3 cells cultured in TIFM in comparison to the cells cultured in RPMI media, showing a decrease in the lipid biosynthesis of these cells in these conditions. However, there also seems to be lower palmitate levels in the TIFM-cultured mPDAC3 cells specifically, in comparison to their mPDAC1 and mPDAC2 counterparts. Why is that? Could the authors comment on this result?

We thank the reviewers for this helpful observation. In Figure 1L (now Figure 1N), we wanted to show how culture conditions (RPMI/TIFM) affected both the total amount of palmitate in PDAC cells but also the fraction that is labeled (i.e. arising from de novo synthesis). We think this provides more information for readers by allowing them to assess both changes in pool size of palmitate and changes in the fraction of palmitate that is synthesized. We like this presentation as it shows clearly that while total palmitate levels behave differently across cell lines (with TIFM culture reducing levels in mPDAC1-2 but increasing levels in mPDAC3) the amount of palmitate that is synthesized de novo is decreased in all three cell lines when cultured in TIFM. To highlight this, we also present the fraction of palmitate that is labeled in Fig. 1O.

We are unsure why TIFM culture reduces total palmitate levels in some PDAC cell lines, while others are able to maintain total palmitate pools. We assume that TIFM cultures increase lipid uptake to compensate for lack of synthesis, and potentially differences in lipid scavenging capacity between the lines could explain this difference. We are currently working on experiments to test these hypotheses and will present the results in a future study.

(6) Figure 2 - Figure Supplement 1A: It would be informative and appreciated to know which nutrients are actually represented and correspond to certain points on the graph, in particular for the ones that are the most differentially present in the two different media.

We have now updated this graph to highlight key metabolites that are most differentially abundant between the two media. We also now provide as a Supplementary file the composition of TIFM, which provides readers with all the information needed to understand which metabolites are differentially abundant in TIFM and any media they wish to compare.

(7) Figure 2 - Related to Figure supplement 1D: It would be useful to know how or why arginine was selected for further investigation from the subset of amino acids. The authors could elaborate on this, by showing or highlighting the data that drew attention to this amino acid initially.

We thank the reviewers for this note. We have tried to make Figure 2 – Figure supplement 1 more clear as to how arginine was selected for further investigation. We have updated the figure to improve clarity for the comparisons of different media that enabled us to identify differences in amino acids between RPMI and TIFM as driving the difference in lipid metabolism. We have also highlighted in Figure 2 – Figure supplement 1A that arginine is the most differentially abundant amino acid and editing the text to explain the logic that this high degree of differential abundance is why we focused on arginine amongst all the amino acids as a likely candidate for regulation of SREBP1.

(8) The legends for Figures 2G and 2H could be improved, i.e., making clearer that 2H shows incorporation in the circulating fatty acids, unlike 2G.

We have updated the figure with improved labeling as the reviewer suggested to denote which panels correspond to which sample type.

(9) Figure 3E and 3G: The heatmaps displayed here show that the addition of arginine to TIFM culture medium restores fatty acid synthesis; however, it appears that the nature of the lipids synthesized in this condition may differ from the ones synthesized in RPMI cultured conditions.

We have added additional text highlighting that arginine supplementation to TIFM and RPMI culture led to induction of different SREBP1-target genes, but that both lead to activation of fatty acid synthesis and desaturation genes, which contributes to the focus of our study on de novo synthesis of saturated and monounsaturated fatty acids in the study.

(10) Figure 3O: The SREBP1 immunoblot still seems to show some residual bands for the cells transduced with SREBP1 targeting sgRNAs, therefore, the authors may want to be more nuanced and present this model as a KD, instead of a KO, as mentioned in the text?

We agree with the reviewer’s suggestion, and we have changed the text to describe these as knockdowns rather than full knockouts.

(11) Figure 3P: Is it possible that there is an error in the legend of the figure (Lipids + for the first bar and - for the second one?). The figure could also be improved by a legend that explains what the different colored bars represent.

We thank the reviewers for pointing out this mistake in the figure and have updated it to correctly label these samples as sgSREBP1 and sgNTG transduced PDAC cell lines.

(12) Figure 4: The authors are stating in Figure 4 - Figure supplement 1A-F, that argininerestricted mPDAC cells are not sensitized to xCT or GPX4 inhibitors that trigger ferroptosis and that therefore SREBP1 suppression by arginine restriction in the TME does not sensitize PDAC cells to ferroptosis inducers. However, this does not appear to be so clear with the data shown. This might be due to the limitations associated with the population doubling measurements instead of the lethality measures noted above. Likewise, later it is proposed that arginine restriction sensitizes both mPDAC cells and human PDAC cells to α-ESA induced ferroptosis. These results would benefit from a direct measure of cell death. Related to the above point, it would be useful to better understand why cells cultured in arginine-deprived TIFM do not appear to be sensitized to ferroptosis inducers, but these same cells die from ferroptosis when treated with α-ESA. It would be useful to present some thoughts.

We thank the reviewers for bringing up this important point. To the reviewers first point, we repeated xCT and GPX4 inhibitor treatment experiments to include both growth corrected (PMID: 27135972) proliferation assays and Sytox-based viability assays. In both cases, we did not find consistent sensitization to xCT or GPX4 inhibitors across multiple PDAC lines when cultured in TIFM. In contrast, we found consistent sensitization to PUFA treatment across multiple murine and human PDAC cell lines cultured in TIFM. Together, this analysis suggests that arginine starvation specifically sensitizes PDAC cells to PUFAs, but not other ferroptosis inducers.

We agree with the reviewer that this is an interesting and unexpected observation. We do not have a mechanistic understanding as to why this is the case. However, we believe this is quite interesting and suggests that PUFAs maybe a better method of inducing ferroptosis in certain conditions than other ferroptosis inducing approaches. We have added text to the discussion to highlight this interesting and unexplained observation.

(13) Figure 6: The authors mention that α-ESA is used here at sublethal doses, which do not affect viability or proliferation, but this is not shown in either the main or supplementary data. These data should be provided somewhere. It might also be nice to mention in the main text (not just in the legend) the dose of α-ESA used for the combination treatments.

We thank the reviewers for this helpful suggestion. To illustrate that α-ESA is used at a sublethal dose, we altered each panel to be on a linear rather than logarithmic x-axis, therefore including the DMSO control arm for each ferroptosis inducer in combination with α-ESA. We hope this now clearly illustrates that this dose α-ESA is not perturbing cell growth or viability in these assays.

(14) Figure 6B: Fer-1 treatment does not seem to rescue the phenotype very clearly. This could again be because cell death is being conflated (to degree) with effects on proliferation, and Fer-1 is not expected to affect cell proliferation. Again, measuring cell death directly would be better than measuring population doublings.

We thank the reviewers for this helpful comment. To address this concern, we have added Sytox-based viability assays to figure 6. These assays indicate that Fer-1 treatment rescues the viability of PDAC cells treated with ferroptosis inducers, α-ESA, or the two in combination.

**Reviewer #3 (Recommendations for the authors):**

General notes:

(1) It would be easier for the reader if one condition were consistently placed in the same position throughout the graphs. For example, RPMI results should always appear first and TIFM second. Currently, this is inconsistent throughout the manuscript (e.g., Figure 1 - Figure Supplement 1: RPMI is first and TIFM second; Figure 2 - Figure Supplement 1: TIFM is first and RPMI second).

We thank the reviewers for this note. We have updated the figures to remain consistent in their ordering throughout the manuscript.

(2) Please briefly explain the differences between PDAC1-3 and clarify why most follow-up experiments were conducted using PDAC1. Presumably, this was because PDAC1 showed the most robust effect on fatty acid synthesis.

We have added additional text in the results section of the manuscript describing the different murine PDAC lines used in this study. We performed most studies with mPDAC1 as this line has robust differences in fatty acid synthesis between culture conditions. However, murine PDAC lines recapitulate the transcriptional subtype diversity of PDAC (PMID: 29364867), so we critically repeat key experiments in multiple mPDAC lines to determine if a given finding is translatable to other PDAC subtypes.

(3) Are only SREBP1 protein levels affected or are SREBP1 RNA levels also decreased in low arginine TME?

We appreciate this important comment. We have added SREBP1 RNA levels to Figure 1 to show that RNA levels do not differ between conditions, whereas protein levels of SREBP1 change significantly.

(4) What was the rationale for investigating lipid metabolism even though it was not the top changed metabolic gene signature? It would be interesting to briefly discuss which pathways were the most enriched.

We chose to focus on lipid metabolism as multiple transcriptomic analysis tools, namely GSEA and GATOM, which specifically focuses on enrichment in KEGG annotated metabolic pathways, highlighted lipid synthesis as being the most transcriptionally regulated metabolic pathway in TIFM. To make this apparent to readers, we added an additional Figure supplement to Figure 1 that includes a comprehensive list of up- and downregulated gene sets in PDAC cells cultured in TIFM from GSEA and GATOM analysis. We hope these additions will make the logic for our focus on lipid synthesis clear and will be useful for readers in highlighting other metabolic pathways that are significantly altered by nutrient stress, such as the TCA cycle and oxidative phosphorylation.

Further comments:

(1) Figure 1 Supplement 1A: It is not clear which SREBP target genes are significant. Please indicate this more clearly.

The analysis in this section was done on expression level of all the indicated genes between groups (tumor/normal) rather testing for significance of individual genes between the two groups. We have updated both the text and the figure legend to clarify this as the statistical analysis that was performed.

(2) Figure 1J and 2C: The Western blot loading control (Actin) does not appear equal across all samples. It would be helpful to include a quantification normalized to the Actin loading control.

We have included quantification of each western blot to help interpret these immunoblots.

(3) Supplementary Figure 2: How often has this experiment been performed? The TIFM results appear to consistently show the same values. If this is the case, it needs to be labeled appropriately.

Thank you for pointing out that how we presented the data was confusing as to how the experiment described was performed. Initially, we performed multiple separate experiments to identify arginine starvation as the TIFM-driver of SREBP1 suppression. To compare across all the separate media conditions, we performed one experiment with all the relevant media conditions together, which is the experiment that is described in the manuscript. Thus, there was one set of control TIFM/RPMI conditions to which we compared all of the different media conditions. As we initially presented the data, it appeared as if we had performed multiple experiments in which the TIFM/RPMI controls had exactly the same behavior, which is not the case. We have updated the data presentation in this figure to make it clear that this was the experimental design for the data presented.

(4) Figure 3P: Please add a legend for this panel.

We thank the reviewers for point out this mistake in the figure and have updated it to correctly label these samples as sgSREBP1 and sgNTG transduced PDAC cell lines.

(5) Figure 4 - Figure Supplement 1: Please review the legend carefully. The legend currently includes only circles, but some of the graphs (A and F) display squares.

Thank you for catching this mistake. We have updated the panels and legends for this figure so they are concordant.

(6) Figure 4D: The effect of a-ESA treatment on the doubling delta of arginine-treated versus non-treated TIFM cells looks similar. It looks like the difference is because cells treated with arginine start at higher doubling values from the beginning. I would suggest looking at the delta and subsequently tone down the statement: "Addition of arginine significantly decreases sensitivity to a-ESA."

Thank you for this helpful comment. To avoid any confounding effects of differences in basal growth rate between mPDAC cells grown in different media, we have converted all of our data to GR values as described in (PMID: 27135972) which enables us to take into account the basal growth rates of cultures when calculating the effects of treatments/perturbations on culture growth and viability. We hope this addition makes the effect that arginine has on α-ESA sensitivity clear beyond the impact that arginine has on basal growth rate.

In addition, we also measured the viability of α-ESA treated mPDAC cells with and without supplemental arginine (current Fig. 5E) by Sytox-exclusion assay. We believe this new data supports the claim that arginine makes PDAC cells resistant to the addition of exogenous PUFAs.
